# Modifiable Areal Unit Problems for Infectious Disease Cases Described in Medicare and Medicaid Claims, 2016–2019

**Published:** 2024-05-13

**Authors:** Nick Williams

**Affiliations:** National Library of Medicine, Lister Hill National Centre for Biomedical Communications, Maryland, United States of America

## Abstract

**Introduction:**

Modifiable Areal Unit Problems are a major source of spatial uncertainty, but their impact on infectious diseases and epidemic detection is unknown.

**Methods:**

CMS claims (2016–2019) which included infectious disease codes learned through Systematized Nomenclature of Medicine Clinical Terms (SNOMED CT) were extracted and analysed at two different units of geography; states and ‘home to work commute extent’ mega regions. Analysis was per member per month. Rolling average above the series median within geography and agent of infection was used to assess peak detection. Spatial random forest was used to assess region segmentation by agent of infection.

**Results:**

Mega-regions produced better peak discovery for most, but not all agents of infection. Variable importance and Gini measures from spatial random forest show agent-location discrimination between states and regions.

**Conclusion:**

Researchers should defend their geographic unit of report used in peer review studies on an agent by-agent basis.

## INTRODUCTION

Understanding when cases of endemic disease are increasing (becoming epidemic) is partly determined by the geographic unit of surveillance and reporting. When geography is the unit of report, cases are aggregated into preconceived places and said aggregates are compared. This aggregation creates a complex form of bias called Modifiable Areal Unit Problem (MAUP) spatial uncertainty [1–3]. MAUP means that when artificial boarders contain terms (numbers), shifts in the boarders create meaningful shifts in statistical conclusions drawn from said terms [4–7]. In public health reporting, states and counties are the most common unit of report. Counties and states are juridical formed objects which follow MAUP terms [8–11]. There is nothing natural, disease specific or ‘epidemic detection friendly’ about using juridical districts for case aggregation and outbreak detection. Most likely state and territorial epidemiologists report cases at jurisdiction level because their decision making and reporting requirements are jurisdiction specific. Yet diseases do not infect jurisdictions; they infect individuals in transmission chains [12–14].

Traditionally population central tendencies for infectious diseases are learned from case reporting and statistical adjustments relative to known errors such as seasonality, non-respondent clinical sites or risk factors (age, vectors). These central tendencies are used for epidemic detection. The resulting rates learned from central tendencies may be mathematically accurate but the resulting action and inaction by health authorities can be inappropriate if the geographic unit of reporting masks or promotes an epidemic departure from endemic levels falsely. This occurs mostly with denominator selection but also may occur with jurisdiction boundaries for MAUP reasons. States in the United States (US) are not naturally formed or seasonally redistricted (unlike census tracts or congressional districts), nor are counties. Rather they are historically formed and do not reflect daily life or transmission dynamics. This paper reports the results of a comparative experiment which uses two geographic reporting standards for the same population over time to detect changes in population central tendencies to express infectious diseases. Static states and natural mega regions learned from ‘home to work address’ ranges offer an artificial to natural human terrain comparison. If a specific disease has a higher acuity in one geographic method than another, disease specific geographic units of report may be more accurate than artificial ones.

## MATERIALS AND METHODS

### Setting and participants

This study considered all Medicare and Medicaid claims from 2016–2019 from the Chronic Conditions Warehouse. Any beneficiary with a populated beneficiary ID from 2016 through 2019 was considered; up to 2% of records lack attributable beneficiary ID in any given data year. Claims were enrolled if they had an infectious disease diagnosis code. Infectious disease diagnosis codes were mapped to agent of infection learned from SNOMED CT to ICD10-CM mapping. Individuals who had infectious diseases that were not mapped through SNOMED CT were not considered; a comprehensive infectious disease extract for ICD10-CM may not yet exist. This study considered 550 infectious disease diagnostic codes from ICD10-CM and 99 causative disease agents learned from SNOMED CT. Infection codes without agents were mapped to ‘not otherwise specified’, or NOS and agents with varying levels of specificity were post fixed NOS if generic level. For example, Hepatitis would return Hepatitis-NOS, Hepatitis-E would return Hepatitis-E as a causative agent. It is possible that a Hepatitis-NOS diagnostic code refers to Hepatitis-E; NOS infections reported below should be interpreted with care if there is diversity within disease dynamics between agents that could be expressed as NOS. There are several ‘infection’ codes which lack an agent, such as ‘ear infection’, ‘viral infection’ or ‘bacterial infection’. These codes were also aggregated as NOS, Viral-NOS or Bacterial-NOS respectively. Towards agent of infection aggregation from diagnostic codes, agents Influenza, Syphilis and Tuberculous subsume several (body) site, severity and sub-lineage specific diagnostic codes and would reduce to Influenza, Syphilis and Tuberculous agents distinct within the claimant-geography-month.

Geography and time was harvested and assigned to a claim diagnosis from demography files which describe individuals with beneficiary IDs and home address. This model should approximate place-disease-case-counts Per Member Per Month (PMPM). Two geographic units of report were used, state of residence or mega-commuter region of residence. The resulting data sets describe the distinct individuals per month billing for the agent of infection by state of residence or commuter district. Home zip code to state unit of report was learned from the US Census zip code to county relationship files for 2010–2020. Mega regions were learned from Nelson et al., *via* commuter distance extents which were used in this study [15]. Home zip codes were fit to Zip Code Tabulated Areas (ZCTA 2010) and intersected with a shape file of mega regions in Q-GIS. Analysis was completed in SAS and R.

### Unit of analysis

This study considers the distinct number of Members (Medicare and Medicaid) Per Month billing a distinct infectious disease diagnosis claim where the disease could be an agent of infection. These PMPM cases were aggregated within geography, month, and year. They were further used as the numerator in a relative rate where the denominator is the total number of distinct members within a geography over the study period. This ‘rate’ is used below as the unit of analysis.

**Analysis 1:** Departure from the central tendency by agent of infection and jurisdiction over time

Across geographic units, rates were evaluated for their moving average departure from the median agent-geography rate. This moving average considered current, post and prior month or three value average. Months where the average rate was above the median within disease and geographic unit were summed and compared by geographic unit of reporting. Higher, ‘above median months’ should mean the fitness of a geographic unit for detection, as high tail PMPM can be detected more often in one geographic unit of reporting and not others.

**Analysis 2:** Segmentation of geographies from infectious disease use cases

Towards the segmentation of mega regions from mega regions and states from states, a spatial random forest model series was produced using spatial ML package in R [16]. The spatial model considered a choice set of diseases and attempted to use the disease claim PMPM divided by the members within a region (case rate) to tell regions apart from one another, as well as tell states apart from states. The models used choice agent PMPM rates as independent variables when considering leaf assignments. The state and region model considered 200 trees per forest. Table 2 presents’ model summary values and figure three describes the geography to disease specific variable importance from the models as an interquartile range.

Segmentation of geography from disease agents can help detail the differences between geographic units of report using real world data and agent specific use cases. Towards interpretation, high model scores does not mean that the case rate was high, but that the specific disease rate was high value in making a prediction about which state or region is associated with which disease. Five nearest (unit of report) neighbours were used to locally weight the geographic evaluation/random forest associations; a rate could be higher than the five nearest geographic units or lower and achieve segmentation from neighbours. Spatial random forest considered all study units of report (states and regions).

## RESULTS

**Figure 1:**
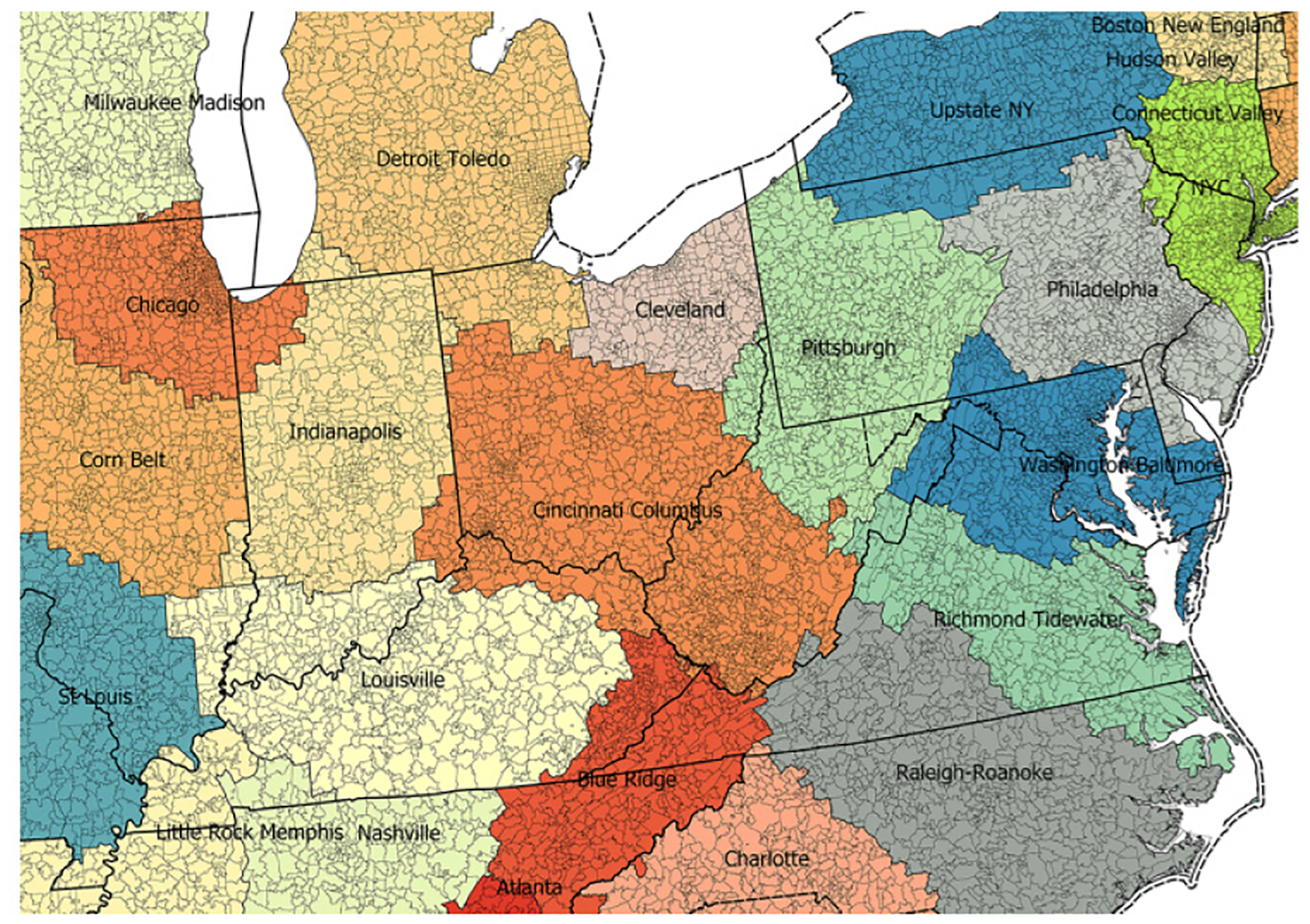
Mega region commuter extents with state outlines and ZCTA detail.

States *vs* mega regions learned through ZCTA is a complex geoprocessing concept and central to this paper. Figure 1 demonstrates this complexity visually with large black lines outlining states, colored, labeled regions demonstrating mega regions and ZCTA contained within as small enclosed black lines. For example the Philadelphia mega region includes ZCTA’s from states Pennsylvania, New Jersey, Maryland, Delaware and New York State. The city of Philadelphia is enclosed in the state of Pennsylvania. The state of Pennsylvania includes ZCTAs from some but not all megaregions: Philadelphia, Pittsburg, Upstate New York, New York City, and Washington-Baltimore mega regions. The mega regions are home to work maximum extents learned through census records (Figure 1).

Eighty six out of ninety-nine infectious agents occurred in regions and states with greater than 11 PMPM and were analyzed. Table 1 tabulates the relative case capture across all study months by agent of infection for states and regions for the 20 highest cumulative PMPM infections. Do note that ‘states’ in CMS includes Puerto Rico, Guam, US Virgin Islands as well as out-of-US region codes for beneficiaries abroad. Differences in table one should be interpreted with caution as there are slightly more regions than states, and total cases presented in table one is uncontrolled for eligible population (from which cases were drawn). Further, cases are distinct individuals who can be discovered once, monthly over four observation years for a maximum of ‘one person to agent to forty-eight case months’ ratio. Individuals who moved (changed their mailing address over the study period and crossed state lines would count twice under State PMPM). Individuals who moved across regions would also count twice should they bill for an agent of infection in the new region. Regions may capture a larger breadth of geographic change over time than states (Table 1).

**Figure 2:**
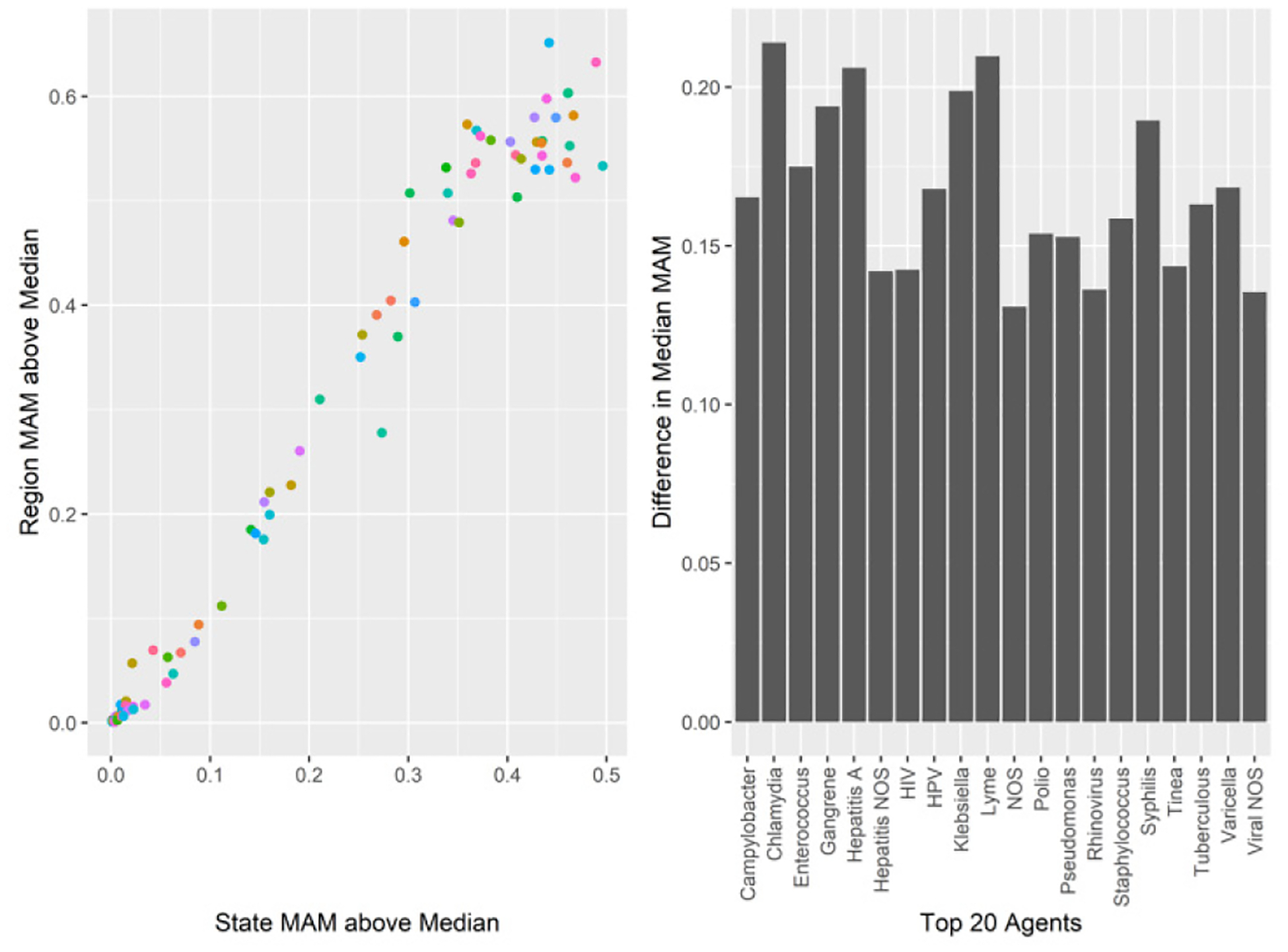
Top 20 PMPM monthly moving average above the median by geography type and agent.

Figure 2 describes the relative difference between the share of states and regions in which the monthly moving average was above the series median. This should produce ‘peak’ detection. High peak months by states and regions are plotted below as the percent of geographic unit-agent-months. Both states and regions had superior detection for specific agents of infection. Note that ‘rare’ diseases are better detected with states than regions. The percent difference between geography ranged from .01% to 21.04% of months.

Within the spatial random forest model non-states (colonies, territories) were not used, Hawaii and Alaska were further excluded as their nearest neighbours are not rational study distances. Washington DC was considered. The segmentation models attempted to guess the geographic unit’s name from a select list of infectious agents and their monthly rates. The model knew the local area of the given unit through nearest neighbour local areas learned from centroid longitude and latitude. The Spatial ML package builds random forest ‘trees’ from the local area of a geographic observation rather than consider the total universe of observations. The models considered 200 trees and choice infectious agents (independent variable) could be used for assignment. The segmentation model was highly accurate, with states error (bad guesses) at 04.89% and regions at 02.65%; denoting that regions were better than states when considering segmentation potential learned from select agents.

Table 2 describes the differences in GINI between the models. Here Mean Decrease GINI (MDG) could be understood as the distinctiveness of the segmentation decisions. The higher the MDG, the more acute the independent variables (disease case month volumes) used to make a split on a tree. For example, in table two syphilis had an increase in MGD between geography types of 113.08, so states used additional information more often when considering syphilis relative to regions. Larger values indicate that a geography is better at finding segmentation using fewer diseases when a specific disease is present in the decision. Note that different geography types consider different diseases when deciding on segmentation.

**Figure 3:**
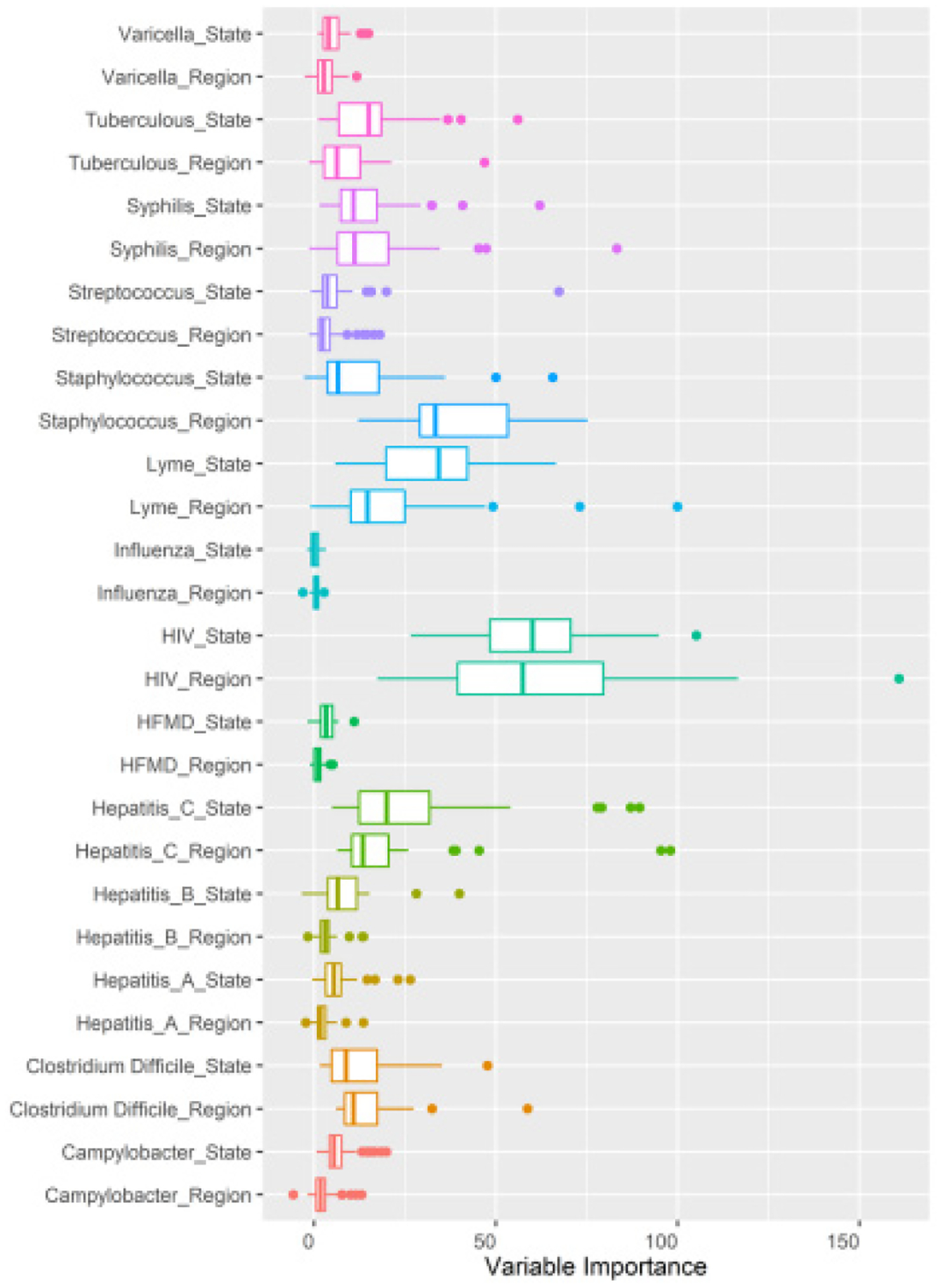
Model variable importance with choice agents.

Figure 3 considers the variable importance by geographic unit of report, which is the contribution the agent of infection made to the segmentation decision when spatial random forest models attempt to tell labelled geographies apart. The difference between variable importance should be understood as the interquartile range of the segmentation of the diseases associated with geographies. Large shifts are detectable within diseases such as HIV which had its largest variable importance for, Region: Miami at 160.83, and State: New York with 105.13. Several general population infections which lack geo-specificity were of low model value, in particularly Hepatitis A and Influenza. Staphylococcus (Regions) and Lyme disease (States) had noticeable departures in range. Larger interquartile ranges may suggest a geographic type’s superior fitness in detecting endemics becoming epidemic.

## DISCUSSION

The key finding from above is that geo-specificity improves detection of MAM above median, and that specific diseases may gain detection from specific geographic units of reporting. Here the denominator (spatial) comparison demonstrates that denominators should be specific, intentional, and defended. Prior work indicates that while the discovery of geo-specificity in human infections is certainly not novel, the attribution of a geographic unit of report to different disease detection sensitivity is an important discovery [17–21]. Most geo-specificity studies do not consider different heredity in their geographic comparisons as is done here. States are juridical and mega regions are learned from daily human behaviour of employment migration. If an infectious disease has ‘specific’ behavioural or vectorised terms of spread custom geographic units of report may improve outbreak detection, control, and eradication efforts. This is generally understood in vector borne disease circles but human to human transmission remains ‘under-vectorized’, especially in terms of a spatial epidemiology which discriminates between illnesses as is done here.

The relationship between risk pooling and natural history could be considered a ‘spatial acuity’ problem. This concept of spatial acuity of disease should be interpreted as the ‘accuracy’ or fitness for use of a unit of report. It should not qualify a unit of report inherently but can demonstrate how a disease has a relationship to populations in places. A representative spatial acuity for a disease agent should be highest of trialed options as above. This paper demonstrates that spatial acuity of disease can be detected, modeled, and compared using current methods. This paper does not demonstrate that the alternative geography, regions, are inherently fit for purpose but does demonstrate on a disease-by-disease basis their comparative fitness. Other administrative (Zip Code Tabulated Areas, census blocks) and natural (commuter district distance bands, home to work address ranges, cell phone GPS tessellations) could be evaluated on a disease by disease basis for relative fitness assessment as units of reporting [19,22].

This study has implications for clinical demography. Consider that New York State (NY) has historically had 10%−12% of the national HIV burden, and only 5%−6% of the national population [23]. By disaggregating New York State into several mega regions and considering multiple diseases in segmenting the regions from one another, Miami-Region has a more ‘deterministic’ burden of HIV illness in this study than the District of Columbia (DC) or NY-State. This is because of the local area, nearest neighbour pass the model is performing before considering tree/leaf assignments. If the New York City-Region is surrounded by similar HIV prevalence regions the spatial acuity of its disease burden will be lower than a region like Miami which is surrounded by low prevalence regions.

Life time infection risk in DC may be as high as one in thirteen people rivalling the worst affected nations [24]. The utility of saying that a jurisdiction has a ‘HIV infection life time expectation’ may be confounded by the natural history of the population which is regionalized (DC-Baltimore Region), segregated (racially, economically) and in the case of Chicago gentrification, migratory [25–29]. In this example HIV is under articulated using the state boundary as a unit of report and a different, perhaps population specific cartography could segment the risk of contracting HIV with more acuity.

Future work should consider an epidemiology which differentiates between the distributions of ‘susceptible’ cases; it would perhaps be the natural extension of this effort. A geographic surface which computed regions as above could, with proper population detail make a more specific segmentation within a disease rather than a spatially determined view of a disease. Further, complex co-infections like HIV, Tuberculosis and Hepatitis C are most likely occurring in similar populations in the US raising the utility of complex spatialized views [30–32].

## CONCLUSION

Intentional geographic units of analysis could improve endemic to epidemic detection. States and mega regions had competitive utility in series segmentation and neither provided a completely superior method for every agent of infection. Future studies should justify their use of geographic unit in infectious disease studies and consider spatial uncertainty in disease detection. Studies and perhaps infection surveillance should use disease specific geographic standards for their unit of surveillance on an agent by agent basis.

## LIMITATIONS

This method assumes that an increase in the index of billing in a geographic area on CMS claims is not irrational; it may well be in some cases. Treatment, novel infection, and incidence cannot be disambiguated readily from prevalence in claims data. The assumption of co-linearity between diagnostics and positive test results over time is not evaluated here either. A ‘true’ case study might consider reportable infections as a unit of analysis rather than claims data but such record systems often report pre-aggregated records rather than the re-identifiable encounter level records use here. Consequently this data set is not a ‘true’ epidemiology of infection in the CMS population as only infections that were billed and mapped through SNOMED CT were considered. Further the quality of ICD10-CM to SNOMED CT mapping to infectious diseases remains under evaluated. Treatment (HIV in particular) was not disambiguated from novel infection. Claims data can describe infectious agents, but these exposures to infectious disease codes could well be justifications for a screening test rather than a ‘true positive’ infection case. In turn the unit of analysis should be understood as the departure from the rolling median for outbreak detection rather than a true case epidemiology.

The denominator of the rate in the above study is simply the available members; infectious diseases are perhaps pickier than this in most real-world scenarios. The susceptibility of a subpopulation to a disease was not considered in calculating the denominator and a cases-to-susceptible rate may produce larger effect sizes than the case-to-beneficiaries rate period presented here. For example, Hand, Foot and Mouth Disease (HFMD) is the providence of small children in population dense settings such as day care centers; all members were considered HFMD eligible in this model. The consequences of this is perhaps an underestimation of the study effect sizes.

The results of this study should be considered an estimate for considering novel units of reporting, rather than a true floor or ceiling of infection burden within the CMS population. Most likely CMS patients bill care to non-CMS payors such as out of pocket or third-party insurance program like Human Resources Service Administration Ryan White, VA, health savings accounts or private insurance. These non-CMS payors should capture some of the infectious disease codes in every data year without sharing charges with CMS. In turn CMS would be none the wiser to the true burden of infection among the population.

## Figures and Tables

**Table 1:** Top 20 infectious agents by case-month and geographic unit.

Agent of infection	State PMPM cases	Region PMPM cases
Tinea (Ring worm)	5,70,32,882	5,99,26,428
*Streptococcus*	1,32,90,331	1,41,70,451
Influenza	1,06,76,429	1,13,35,067
Viral NOS	76,49,741	80,31,743
Candida	71,25,179	75,26,649
HIV	61,62,992	62,85,040
Hepatitis C	39,60,574	41,53,671
Clostridium Difficile	22,71,677	24,00,716
Hand Foot (and) Mouth Disease (HFMD)	20,65,127	21,60,636
Syncytial Virus	17,22,965	18,42,686
*Staphylococcus*	17,00,042	18,04,160
Molluscum Contagiosum	12,47,306	13,05,903
Mycosis NOS	12,27,030	12,94,095
*Pseudomonas*	11,61,381	12,30,902
Bacteria NOS	9,98,400	10,48,788
Lyme Disease	8,12,570	8,73,648
Hepatitis NOS	6,50,807	6,75,578
Blast mycosis	4,47,016	4,74,869
Infection NOS	4,46,024	4,71,091
HERPES NOS	4,36,373	4,55,989

**Table 2:** Mean decrease GINI by region and state, spatial random forest model with choice agents.

Mean decrease GINI	Region	State	Difference
HIV	695.86	409.54	286.33
*Staphylococcus*	411.54	167.36	244.18
Syphilis	341.71	228.62	113.08
Lyme	304.77	233.27	71.51
Clostridium difficile	255.92	228.74	27.18
Hepatitis C	255.65	329.38	−73.74
Tuberculous	120.69	239.66	−118.97
*Streptococcus*	53.39	85.43	−32.04
Hepatitis B	44.19	102.27	−58.08
Varicella	40.68	54.31	−13.63
Hepatitis A	28.03	83.42	−55.39
Campylobacter	25.59	96.21	−70.62
Hand foot and Mouth disease	9.43	35.69	−26.26
Influenza	3.56	9.12	−5.56
